# Weight Excess in Developmental Age: Health Outcomes Through a Socioeconomic Lens

**DOI:** 10.3390/nu18203414

**Published:** 2026-10-18

**Authors:** Maria Elena Capra, Leonardo Detto, Giada Sica, Ada Marcella Chiesa, Susanna Esposito, Giacomo Biasucci

**Affiliations:** 1 Pediatrics and Neonatology Unit, Guglielmo da Saliceto Hospital, Piacenza 29121, Italy; m.capra@ausl.pc.it (M.E.C.); giacomo.biasucci@unipr.it (G.B.); 2 Department of Medicine and Surgery, University of Parma, Parma 43126, Italy; 3 Pediatric Clinic, University Hospital of Parma, Parma 43126, Italy

**Keywords:** obesity, overweight, childhood, adolescence, socioeconomic burden, non-communicable diseases

## Abstract

Childhood overweight and obesity are escalating global health challenges with consequences that extend across the life course. This narrative review aims to provide a comprehensive overview of the implications and consequences of excess weight in children and adolescents across different socio-economic settings, analyzing weight excess-related health outcomes through a socio-economic lens. Evidence was identified through searches of MEDLINE (via PubMed), Scopus, Web of Science, Embase, and the Cochrane Library, supplemented by manual screening of reference lists. The literature indicates that excess adiposity, particularly visceral fat, promotes insulin resistance, hypertension, dyslipidemia, hepatic steatosis, and other early manifestations of cardiometabolic disease, while also contributing to respiratory, orthopedic, and psychosocial complications. These risks frequently persist into adulthood, increasing premature morbidity, mortality, healthcare utilization, and direct and indirect societal costs. The burden is unevenly distributed: poverty, stigma, discrimination, food and built environments, and limited access to preventive and clinical services shape both obesity risk and outcomes, with particularly severe consequences in resource-constrained settings. Early identification of excess weight and associated risk factors, systematic screening for comorbidities, and timely, family-centered multidisciplinary care are therefore essential. Effective prevention and treatment must also address the broader social and commercial determinants of health through coordinated action across healthcare, education, food systems, and public policy.

## 1. Introduction

Overweight and obesity are among the most widespread chronic conditions in childhood. According to American Academy of Pediatrics (AAP) guidelines, obesity is a complex chronic disease characterized by excess adiposity resulting from the interaction of genetic, physiological, behavioral, and environmental factors within an increasingly obesogenic environment [1]. The clinical definition is based on the Body Mass Index (BMI), with overweight defined as a BMI between the 85th and 95th percentile and obesity as a BMI above the 95th percentile for age and sex. In preschool-aged children and infants, weight–length z-scores are used, whereas additional tools such as the waist-to-height ratio help assess central adiposity, a marker strongly linked to cardiometabolic risk [2]. Pediatric obesity is the leading risk factor for several non-communicable diseases (NCDs). Excess adiposity, particularly visceral fat, is associated with early development of insulin resistance, hypertension, dyslipidemia, and hepatic steatosis, all of which contribute to metabolic syndrome and, over time, to cardiovascular diseases and type 2 diabetes. Evidence from the Italian Society for Pediatric Endocrinology and Diabetology shows that early-onset obesity—especially before the age of 5—prolongs exposure to cardiometabolic risk factors, promoting adiposity tracking and early subclinical cardiovascular damage [3]. Weight excess is classified as an independent cardiometabolic risk factor, and subjects who are overweight or obese should be considered at increased cardiovascular risk [4,5]. Longitudinal studies have demonstrated that children in the highest BMI quartile have more than double the risk of premature death, including cardiovascular mortality, compared with their normal-weight peers. Obesity’s impact extends beyond metabolic risks. Recently, the global rise in childhood obesity has been closely linked to increases in hypertension, type 2 diabetes, non-alcoholic fatty liver disease (NAFLD), metabolic dysfunction-associated fatty liver disease (MAFLD), respiratory diseases, and psychological disorders, which now constitute a significant burden of non-communicable diseases among children and adolescents [6]. Moreover, notable geographical and socioeconomic disparities emerge: middle-and low-income countries show higher prevalences of hypertension, metabolic syndrome, and NAFLD or MAFLD among children with obesity; boys are generally more affected than girls; and the highest rates are often reported in Asian populations. These disparities reflect the influence of social determinants of health—such as poverty, limited access to care, stigma, and discrimination—which contribute both to the development of obesity and to the complexity of its clinical course, as also emphasized by the AAP guidelines [7]. Overall, overweight and obesity in childhood represent conditions with substantial biological, clinical, and social impact, with consequences that extend from childhood into adulthood. In this context, with the publication of Law No. 149 of 3 October 2025: Disposizioni per la prevenzione e la cura dell’obesità (25G00158) (GU Serie Generale n.235 del 9 October 2025), Italy, becomes the first country in Europe to recognize obesity as a chronic, progressive, and relapsing disease with significant social and health impacts and establishes integrated interventions for prevention, diagnosis, and treatment to improve patients’ quality of life. Early identification of excess weight and related risk factors, together with systematic screening for comorbidities, is essential to prevent non-communicable diseases and safeguard long-term health in children and adolescents. This narrative review aims to provide a comprehensive overview of the implications and consequences of excess weight in children and adolescents across different socioeconomic settings, analyzing weight excess-related health outcomes through a socioeconomic lens.

## 2. Materials and Methods

We conducted a literature search in MEDLINE (via PubMed), Scopus, Web of Science, Embase, and the Cochrane Library to identify relevant publications published between 2005 and 2026.

This narrative review used a structured, transparent literature search. No a priori protocol was registered, as this review was intended as a narrative synthesis rather than a systematic review. Nevertheless, we predefined the main steps of the search and selection process to ensure transparency and reproducibility. The search strategy combined terms related to excess weight and developmental age (“obesity” OR “overweight”) AND (“childhood” OR “adolescence” OR “developmental age”)—with terms describing health and socioeconomic outcomes, including “non-communicable diseases,” “health consequences,” “dietary patterns,” “health outcomes,” “outcome measures,” “socioeconomic status,” “socioeconomic burden,” and “global burden.” The search aimed to identify randomized controlled trials, peer-reviewed observational studies, systematic reviews, meta-analyses, umbrella reviews, and comprehensive narrative reviews relevant to this article’s scope. The last search was conducted on 30 August 2026.

No database-level filters were applied during the initial search. Publications were eligible if they were available in full text, written in English, published between 2005 and 2026, and examined the prevalence, determinants, health consequences, management, socioeconomic burden, or socioeconomic inequalities of overweight and obesity in children or adolescents across different social and geographic contexts. Studies published before 2005 (with a few exceptions for older seminal or contextual publications that were permitted outside the primary search strategy), articles not available in English, and publications outside the predefined population, topic, or study categories were excluded. The literature search was limited to studies on children and adolescents, even if some cohorts included young adults.

Two reviewers independently screened the titles and abstracts of the retrieved records and subsequently assessed the full texts of potentially relevant publications. The reviewers resolved any disagreements about eligibility through discussion and, when necessary, consultation with a third reviewer. After removing duplicates, we evaluated each article based on its title and abstract. We excluded articles that were background or unrelated studies, addressed an unsuitable dietary exposure or demographic, were an inappropriate publication type, reported incorrect outcomes, or were published in a language other than English. We then conducted full-text assessments and resolved any eligibility discrepancies by discussing them with a third author. To identify additional pertinent studies, we also manually examined the reference lists of the selected articles. The structured literature search identified 1650 articles published between 2005 and 2026. After removing duplicates, we screened 670 records by title, 250 by abstract, 90 in full text, and ultimately included 50 in the review.

As this was a selective narrative review, we prioritized large, methodologically robust randomized controlled trials; peer-reviewed observational studies; systematic reviews; meta-analyses; umbrella reviews; and authoritative narrative reviews. The authors did not conduct a formal systematic-review protocol, quantitative evidence synthesis, or standardized risk-of-bias assessment.

## 3. Weight Excess and Obesity in Childhood and Adolescence

### 3.1. Weight Excess and Obesity Prevalence

The Global Burden of Disease (GBD) 2021 project examines trends over more than three decades, from 1990 to 2021, and offers forecasts through 2050. The central aim of the analysis is to clarify how the prevalence of excess weight is evolving globally and what future burden we can expect if current trajectories continue [8].

A major finding of the study is the dramatic rise in overweight and obesity among individuals aged 5–24 years. Between 1990 and 2021, the combined prevalence of overweight and obesity approximately doubled across this age group, while obesity alone tripled. These changes reflect both the broad expansion of obesogenic environments and insufficient global policy action. By 2021, an estimated 493 million young people worldwide were affected by overweight or obesity. Among them, 174 million were living with obesity, split relatively evenly between children and young adolescents (5–14 years) and older adolescents and young adults (15–24 years). These numbers represent not only a widespread public-health issue, but also a failure to protect a generation now exposed to elevated risks of lifelong metabolic disease. The highest levels of obesity in 2021 were found in North Africa and the Middle East, where many countries showed obesity rates in youths exceeding those of nearly all other global regions. Particularly striking was the high prevalence among adolescent girls, which the authors associate with rapid nutritional transitions, urbanization, reduced physical activity opportunities, and cultural norms around gender. Other regions with very high current prevalence include Australasia and high-income North America, where several countries have already reached an “obesity-predominant” stage, meaning that obesity has surpassed overweight as the main contributor to excess body weight. At the same time, obesity prevalence grew fastest over the past three decades in regions traditionally considered low risk: Southeast Asia, East Asia, and Oceania. For example, some East Asian countries experienced a more than 600% increase in obesity among children and adolescents from 1990 to 2021. These findings underscore that the obesity epidemic is no longer confined to high-income nations, but instead, it has become a global phenomenon affecting countries at all income levels. The authors emphasize a key concept: the global transition from overweight to obesity dominance. Historically, overweight was more common than obesity in youth populations. However, trends now show that in several world regions, including parts of Latin America, the Middle East, Oceania, and high-income countries, obesity has become the primary excess-weight category. This indicates that not only are more young people becoming heavier, but the degree of excess weight is intensifying. Such a shift signals more severe long-term health consequences, given the strong associations between childhood obesity and early onset of metabolic and cardiovascular disease [8,9,10,11]

Using advanced ensemble modeling, the study [8] estimates how young-people obesity will evolve by 2050 if no substantial interventions take place. The projections are sobering; in fact, obesity prevalence is expected to increase in every region of the world by 2050. By mid-century, an estimated 361 million young people aged 5–24 years will be living with obesity; among boys aged 5–14 years, obesity is projected to overtake overweight globally. Moreover, the most substantial future increases are expected in Southeast Asia, East Asia, Oceania, and South Asia

Conversely, overweight alone is projected to decline in some regions or remain relatively stable, not because the problem is resolving, but because more individuals will move from overweight into obesity. This reflects worsening severity rather than improvement.

These findings provide evidence of a persistent, escalating global health crisis. Despite decades of recommendations and targeted interventions, no region is on track to reduce childhood or adolescent obesity by 2030, and many will face accelerating increases.

They attribute these trends to multiple factors, including:

- Rapid urbanization and proliferation of ultra-processed foods
- Increased sedentary behavior and reduced opportunities for physical activity
- Socioeconomic inequalities that limit access to healthy foods and safe environments
- Insufficient political will and fragmented global public-health strategies

The consequences are profound: rising rates of type 2 diabetes, hypertension, non-alcoholic fatty liver disease, and early cardiovascular complications in young people. Unless decisive, multisectoral actions are taken, spanning food systems, urban planning, education, and healthcare, countries will face a substantial and potentially irreversible burden of chronic disease in future adult populations.

Recent data from the national Italian surveillance system OKkio alla Salute (Italian National Institute of Health) indicate that, in 2023, 19% of children aged 8–9 years were overweight and 9.8% were obese, including 2.6% with severe obesity [12].

Since the first national survey in 2008–2009, a modest decline has been observed—overweight decreased from approximately 23% to 19% and obesity from ~12% to 9.8%—although a plateau has emerged in recent years [13].

Despite these improvements, Italy remains among the European countries with the highest prevalence of excess body weight in childhood, underlining the need for robust preventive and therapeutic strategies.

### 3.2. Socioeconomic Lens

Socioeconomic factors—assessed through household income, community resources, and parental education—define the limits of what families can afford to eat in various ways [14,15]:

- The Cost of Health: Whole foods, lean proteins, and fresh produce frequently cost much more per calorie compared to heavily processed, energy-dense options. Low-income families often have to prioritize caloric density over nutritional quality to address food insecurity.
- Reduced educational levels and working various or nontraditional shifts can limit a parent’s ability to monitor screen time, prepare meals at home, or control eating habits.
- Psychological Stressors: Economic hardship acts as a constant trigger for family pressure. Research indicates that emotional distress and stress in early childhood can result in “junk food self-medication,” wherein high-calorie, sugary foods are employed as a way to alleviate psychological discomfort.

The contemporary food environment has shifted into an obesogenic setting—one that encourages weight gain by offering and promoting unhealthy food options. Researchers assess this effect using four essential dimensions, the so-called “4A Framework”: availability, accessibility, affordability, and appeal. The effect of the “4A Framework” on weight excess in pediatric subjects is summarized in Table 1 [14,15,16].

### 3.3. Clinical Risks and Early Consequences

Childhood overweight and obesity are not benign or transient conditions. Excess adiposity in early life is associated with a broad spectrum of metabolic, cardiovascular, hepatic, orthopedic, respiratory, psychological, and social complications [17,18].

In this review, we focused on complications that may more strongly affect later-life non-communicable disease development. Common comorbidities include insulin resistance, dysglycemia, dyslipidemia, hypertension, and NAFLD. NAFLD is a condition related to excess weight that can be identified as early as childhood. NAFLD is characterized by an abnormal buildup of lipids in the liver among individuals without concurrent liver disease and without declared high alcohol consumption (under 30 g daily for men and under 20 g daily for women) [19]. This definition has recently been revised for adults, transitioning from the term NAFLD to metabolic dysfunction-associated fatty liver disease (MAFLD) [20]. A global group of specialists has recently suggested diagnostic criteria for MAFLD in children, but agreement has not been established yet [21]. Children with obesity also face an elevated likelihood of developing type 2 diabetes and metabolic syndrome during adolescence or early adulthood, and they are considered at increased cardiovascular risk [22].

Psychosocial consequences—including low self-esteem, social withdrawal, stigma, impaired quality of life, and mental health difficulties—are likewise frequently reported and may substantially affect wellbeing and developmental trajectories [23,24].

### 3.4. Long-Term Outcomes and Socioeconomic Burden

A large body of evidence indicates that pediatric overweight and obesity significantly increase the risk of premature mortality and cardiometabolic morbidity in adulthood. A major systematic review reported that childhood or adolescent obesity was associated with increased early mortality in four of five cohort studies, and with higher cardiometabolic disease incidence across all included analyses, with hazard ratios ranging from 1.1 to 5.1 [25].

However, BMI tracking from childhood to adulthood modulates this relationship. Individuals who attain a normal weight by adulthood may experience a markedly attenuated long-term risk, suggesting that the adverse consequences of childhood obesity are not irreversible and can be mitigated through timely intervention [26].

Recent European cohort data also demonstrate that adolescent obesity is associated with higher healthcare utilization in early adulthood, reflecting the substantial clinical and economic burden of early-onset obesity [27].

From a public health perspective, overweight and obesity account for significant direct healthcare costs, increased hospitalizations, long-term management of metabolic disease, and expanded use of medical resources. Indirect costs—including productivity losses, absenteeism, and reduced workforce participation—further contribute to the economic impact. Analyses from international and Italian contexts consistently show that pediatric obesity represents a major challenge for health-system sustainability [28].

### 3.5. Early Diagnosis and Management

There is broad consensus that early identification of at-risk children is critical to pediatric practice. The use of BMI and weight-for-length percentiles provides:

1. Objective detection of overweight, obesity, and risk conditions, even in asymptomatic children.
2. Longitudinal monitoring, enabling early recognition of deviations from optimal growth trajectories.
3. Timely implementation of interventions, including lifestyle modifications, nutritional counseling, structured physical activity, and family-focused strategies.
4. Prevention of persistence of obesity into adulthood, thereby reducing the long-term burden of chronic disease.
5. A multidisciplinary framework, reflecting the multifactorial determinants of obesity (genetic, behavioral, environmental, and socioeconomic) and involving families, schools, communities, and healthcare systems.

Within this framework, pediatric obesity must be regarded as a clinical condition with immediate and long-term health implications, requiring proactive and coordinated intervention [1,29,30,31,32,33].

Table 2 summarizes key strategies for early assessment and management of pediatric obesity, highlighting their clinical objectives and relevance across socioeconomic contexts, and Table 3 describes socioeconomic patterns related to excess weight.

## 4. Complications of Obesity in Childhood

### 4.1. Cardiovascular

Childhood obesity initiates and accelerates a constellation of cardiometabolic derangements that increase lifetime cardiovascular disease (CVD) risk. Excess adiposity in children is associated with early development of hypertension, dyslipidemia (elevated triglycerides, low HDL), insulin resistance, and subclinical atherosclerosis (increased carotid intima-media thickness, endothelial dysfunction), all of which aggregate to increase lifetime CVD burden [1,5,29,37,38]. Mechanistically, adipose tissue dysfunction in obesity promotes a chronic, low-grade inflammatory milieu (elevated CRP, proinflammatory cytokines), dysregulated adipokine signaling (leptin resistance, relative adiponectin deficiency), and ectopic fat deposition (pericardial and visceral) that impair vascular homeostasis and myocardial metabolism. These perturbations map onto the recently articulated cardiovascular–kidney–metabolic (CKM) syndrome, which conceptualizes the shared pathophysiology and bidirectional risk amplification among CVD, chronic kidney disease, and metabolic disorders; childhood obesity therefore contributes to the early life course of CKM risk [39].

Clinical consequence and progression: obese children display higher rates of prehypertension/hypertension and early lipid abnormalities compared with normal-weight peers; without intervention, these factors persist into adolescence and adulthood and predict accelerated atherosclerotic disease and heart failure risk. Early identification of adverse cardiovascular phenotypes (e.g., metabolically unhealthy obesity, high visceral adiposity) refines risk stratification beyond BMI alone [39,40,41].

### 4.2. Metabolic

Obesity in childhood is the principal risk factor for the development of insulin resistance and type 2 diabetes mellitus (T2DM) in youth. Adipose-driven insulin resistance arises from ectopic lipid accumulation in liver and muscle, inflammatory cytokine signaling, and mitochondrial dysfunction, leading to compensatory hyperinsulinemia and progressive β-cell stress. Different obesity phenotypes (metabolically healthy vs. unhealthy obesity; visceral vs. peripheral adiposity) convey markedly different trajectories: children with metabolically unhealthy obesity have a substantially elevated risk of early dysglycaemia and progression to T2DM. The interplay between obesity phenotype and diabetes also amplifies cardiovascular risk through shared mechanisms (atherogenic dyslipidemia, hypertension, prothrombotic state) [42,43].

Clinical implications: early screening for impaired fasting glucose, abnormal oral glucose tolerance, and HbA1c in obese youth—particularly those with additional metabolic syndrome features—is critical because glycemic dysregulation in childhood predicts persistent metabolic disease and vascular complications in adulthood. Lifestyle interventions that reduce visceral adiposity and improve insulin sensitivity in pediatric populations demonstrably lower short-term metabolic risk and may alter long-term trajectory [43].

### 4.3. Neurological

Although neuropathy is classically associated with long-standing diabetes in adults, mounting evidence links components of the metabolic syndrome, including obesity, dyslipidemia, and prediabetes, to peripheral nerve injury. Studies indicate that metabolic syndrome (MetS) increases the risk of small-fiber and large-fiber peripheral neuropathy independently of frank diabetes; proposed mechanisms include lipid-mediated neuronal injury, microvascular dysfunction in the vasa nervorum, chronic systemic inflammation, and mitochondrial stress within peripheral neurons. In the pediatric setting, prolonged exposure to obesity and metabolic dysfunction during critical developmental windows may predispose to earlier onset of neuropathic changes, and autonomic dysfunction (orthostatic intolerance, impaired heart-rate variability) has been reported in obese adolescents. Exercise-based lifestyle interventions and targeted metabolic risk reduction show promise in halting or reversing early neuropathic changes associated with MetS [44]. However, studies in this field are limited and based on adult populations; further pediatric studies are needed to better analyze this relationship.

Clinical note: screening for symptoms (paresthesia, numbness), objective sensory testing (monofilament, vibration), and autonomic function where indicated can detect early neuropathic involvement in at-risk obese youth; lipid control and weight reduction are plausible disease-modifying strategies [45].

### 4.4. Dermatological

Obesity in children is associated with an increased prevalence and greater severity of several dermatological conditions, notably psoriasis. Limited emerging evidence suggests that psoriasis co-occurs with obesity, likely reflecting shared inflammatory pathways (Th17/IL-23 axis, systemic cytokines) and clustering of cardiometabolic comorbidities. Obese pediatric patients with psoriasis tend to exhibit more severe disease and higher prevalence of psoriatic arthritis and cardiometabolic comorbidities than non-obese peers. The epidemiologic links are bidirectional: psoriasis is associated with higher odds of obesity and metabolic syndrome, and adiposity exacerbates cutaneous inflammation. Therefore, management of pediatric psoriasis should integrate metabolic screening and lifestyle interventions as part of a comprehensive approach [46,47,48,49].

Practical implication: dermatologic assessment in obese children should include evaluation for signs of systemic inflammation and referral pathways for cardiometabolic risk assessment; weight reduction has been associated with improvements in psoriasis severity in adult studies and is a rational adjunctive strategy in pediatric care [48,49]. However, further pediatric studies are needed to better analyze this relationship.

**Figure 1:**
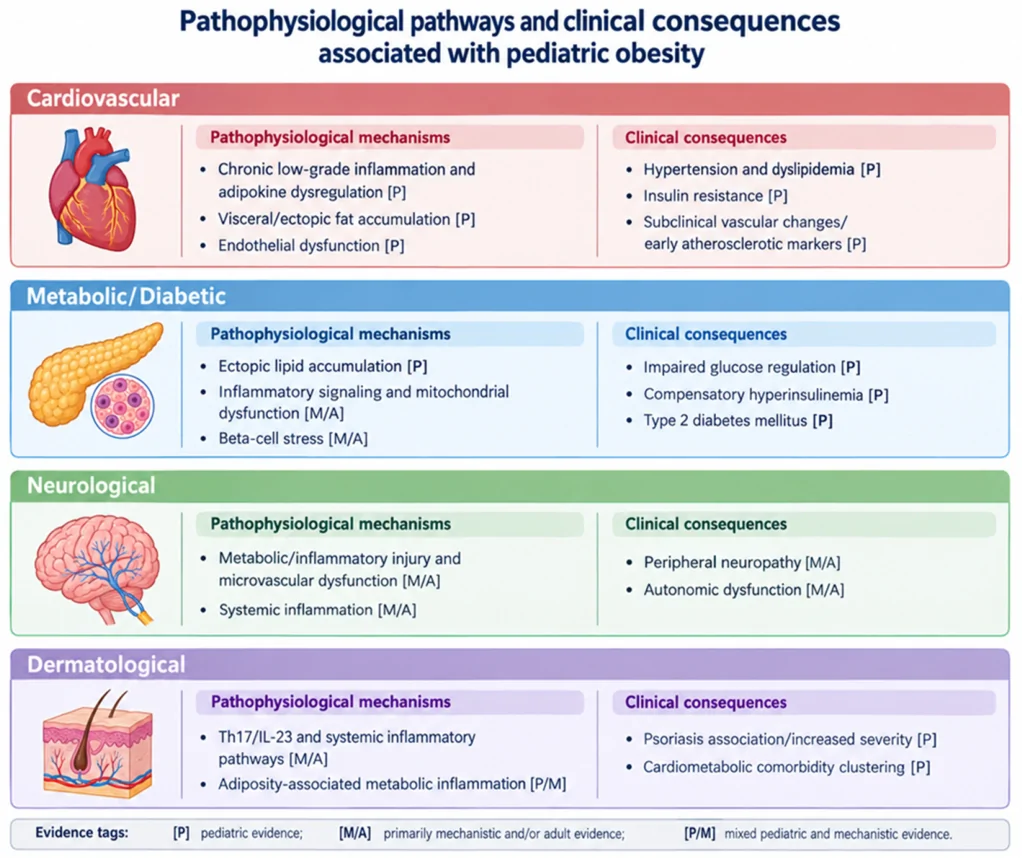
Key Pathophysiological Mechanisms and Selected Clinical Consequences (adapted from [50,51,52,53].

Figure 1 provides an integrated overview of the principal pathophysiological mechanisms linking childhood obesity to selected clinical complications.

In Table 4 we have summarized the main topics debated in our manuscript (adapted from [5,8,12,13,15,39,54,55,56,57,58,59,60,61].

### 4.5. Integrative Considerations and Recommendations

In a recent statement issued by the AHA, recommendations were made on weight excess management in pediatric subjects, in particular:

1. Phenotype-guided risk stratification: BMI alone inadequately captures risk; assessment of metabolic health (lipids, blood pressure, glycemia), fat distribution (when available), and inflammatory markers better identify children at highest risk for cardiometabolic, neurological, and dermatologic complications [45].
2. Early, multi-domain screening: obese children should undergo periodic screening for blood pressure abnormalities, dyslipidemia, glycemic impairment, and targeted neurological/dermatological evaluation when symptoms or risk features are present
3. Mechanism-based interventions: interventions that reduce visceral adiposity, improve insulin sensitivity, and attenuate systemic inflammation (dietary modification, physical activity) [39].

## 5. Socioeconomic Status and Childhood Obesity in Low- and Middle-Income Countries

Childhood and adolescent obesity have increased rapidly worldwide, including in low- and middle-income countries (LMICs). The socioeconomic gradient of pediatric obesity is context-dependent and evolving in the poorest settings; obesity commonly concentrates among higher socioeconomic status (SES) children, whereas with economic development the burden often spreads across and ultimately shifts toward lower-SES groups. This review summarizes contemporary evidence on SES–pediatric obesity relationships in LMICs, highlights regional and gender differences, and outlines implications for research and policy.

Rising prevalence of overweight and obesity among children and adolescents poses major future burdens of cardiometabolic disease. Understanding how obesity is distributed across socioeconomic strata in LMICs is essential to equitable prevention strategies. Pediatric patterns frequently differ from adult patterns because of distinct determinants (early life nutrition, parental SES, school food environments, marketing to children) and because childhood is a critical window for life-course risk [43,54].

### 5.1. Global Trend in Childhood Obesity

Large pooled analyses and global reports document a marked increase in childhood obesity since 1990. Recent NCD-Risk Factor Collaboration estimates indicate substantial rises in obesity prevalence among children and adolescents between 1990 and 2022, with pronounced increases in many LMIC regions. UNICEF and allied analyses now report that, for the first time, more children are obese than underweight at a global level—a sign of the rapid nutrition transition affecting many developing countries.

Systematic reviews focused on children find a complex, context-sensitive relationship between SES and childhood overweight/obesity:

- Low-income settings: In many of the poorest LMIC contexts, studies consistently report a positive association between family wealth/parental education and childhood obesity—i.e., children from wealthier households or with more educated parents are more likely to be overweight or obese. This pattern is frequently attributed to greater access to calorie-dense foods, greater ownership of motorized transport (reduced physical activity), and urban residence [55,56,57,58].
- Middle-income settings: As countries undergo economic development and the food environment changes (greater availability and lower price of ultra-processed foods), the SES gradient becomes heterogeneous. Some middle-income countries display a weakening or reversal of the SES gradient, particularly for adolescent girls, such that lower-SES children begin to exhibit equal or higher prevalence of obesity. This transition often occurs earlier and more strongly among females [59].
- Regional variation: Systematic evidence from sub-Saharan Africa indicates that the positive SES–obesity link remains common in many low-income countries in the region, whereas lower-middle-income countries show mixed associations. Latin America and parts of Asia have documented rapid shifts with growing burdens among lower-SES children in urban settings [57,60].

### 5.2. Mechanisms and Mediators Specific to Children

Several pathways explain the SES–obesity nexus in children: parental resources influence diet quality (both direction and caloric density), early growth patterns (including stunting followed by rapid weight gain), exposure to marketing of unhealthy foods, school food environments, and opportunities for physical activity. Household food security and food affordability interact with cultural norms: in some settings, higher weight is perceived as desirable or a marker of prosperity, which can influence parental feeding practices [61,62].

### 5.3. Public Health and Policy Implications

- Contextualized interventions: Policies must be tailored to the stage of the nutrition transition. In low-income settings, interventions that promote healthy child feeding practices among wealthier urban families (and prevent early-life overnutrition) remain important. In middle-income countries, policies must also target poor and middle-class communities that are increasingly exposed to obesogenic environments [63,64].
- Protecting children from unhealthy food environments: Regulatory actions that limit marketing of ultra-processed foods to children, improve school food standards, and implement fiscal measures (taxes on sugar-sweetened beverages) can protect children across SES groups. These measures are particularly important in LMICs where aggressive marketing and rapid retail penetration have widened exposure to unhealthy products [63,64].
- Life-course and equity lens: Early-life interventions (maternal nutrition, breastfeeding support, prevention of rapid weight gain after stunting) can reduce lifelong obesity risk. Because the burden increasingly affects disadvantaged groups in many LMICs, equity-oriented monitoring and targeting are essential to avoid widening health disparities [61,64].
- Strengthen surveillance and disaggregated data: National surveillance systems should report childhood obesity by SES indicators (household wealth quintile, parental education, urban/rural), sex and age bands. This disaggregation is critical to detect emerging shifts and to evaluate the equity impact of interventions [65,66].

Pediatric obesity in LMICs is increasing rapidly, and the socioeconomic patterning is dynamic: while wealthier families in low-income contexts still often show higher childhood obesity, economic development and pervasive changes in food systems are shifting the burden toward lower-SES children in many middle-income countries. Policies must be anticipatory, equity-focused, and tailored to local stages of the nutrition transition to protect children and prevent widening health inequalities.

## 6. Dietary Patterns

### Epidemiological and Nutrition Transition

The phenomenon observed in many LMICs stems from profound shifts in dietary patterns. A transition has occurred from traditional diets, typically rich in cereals, fiber, vegetables, and minimally processed foods, to a “Western-style” dietary model characterized by high energy density and substantial intakes of ultra-processed products, sugars, and fats, particularly saturated fats derived from animal sources [67,68].

These emerging dietary patterns reflect a shift in habitual food consumption toward regimes dominated by pre-packaged snacks, ready-to-eat meals, and inexpensive, calorie-dense products, often referred to as “cheap calories.” These foods are increasingly accessible, both logistically and economically, and convenient to consume outside the home, requiring minimal preparation [69,70].

In many settings, this shift has resulted in diets that are poor or entirely lacking in legumes, whole grains, vegetables, dietary fiber, and micronutrients, while being disproportionately high in fats, refined carbohydrates, and sodium. Regions historically burdened by chronic undernutrition and insufficient caloric intake have seen increases in per-capita energy availability; however, this increase has not improved diet quality.

The nutrition transition occurs alongside demographic and economic transformations, rapid urbanization, and lifestyle changes [68,69,70,71].

Socioeconomic conditions, food environments, and pediatric health form a continuous cycle. Economic limitations shape the environments where children live, influencing what they eat, impacting their weight, and creating long-term health and financial consequences that persist into adulthood. When the surrounding environment makes unhealthy food the most accessible, affordable, and heavily marketed option, children’s dietary habits adapt accordingly. Nutrient Disparity develops when children in disadvantaged food environments experience high dietary exposure to ultra-processed foods, sugar-sweetened beverages, and trans fats, while lacking adequate fiber, vitamins, and minerals, with a consequent caloric surplus linked to the high palatability and low satiety (fullness) of processed foods, thus easily leading to chronic overeating. This continuous exposure to high-calorie, low-nutrient diets shifts the energy balance, leading to rapid weight gain and increased rates of pediatric overweight and obesity. The impacts of pediatric excess weight extend far beyond health, translating into severe economic and social disadvantages that reinforce the cycle of poverty. Health complications and mental health struggles can lead to higher school absenteeism, lower academic performance, and reduced rates of higher education attainment. Healthcare costs increase, as families face immediate financial strain from managing chronic conditions (such as medications, specialist visits, and hospitalizations). Moreover, adults who experienced childhood obesity face statistically lower wages, reduced workforce productivity, and higher rates of early disability. This economic penalty makes it difficult to escape the low socioeconomic conditions that started the cycle [72,73,74].

This epidemiological shift mirrors patterns documented in previous periods of accelerated development. While the burden of infectious diseases remains substantial, NCDs are rising sharply in LMICs. This trend largely reflects modifiable risk factors.

A growing body of evidence documents increasing rates of overweight and obesity among children and adolescents in low- and middle-income regions. In several Latin American and Asian countries, the prevalence of pediatric overweight has reached alarming levels. Systematic reviews focusing on developing Asian countries highlight dietary profiles marked by frequent fast-food consumption, sugar-sweetened beverages, and commercially packaged snacks, along with low fiber intake and high meat consumption, patterns consistently associated with overweight and obesity.

This evolving dietary model is further reinforced by demographic aging, declining levels of physical activity, increasingly sedentary occupations, and the widespread shift toward urban living. Collectively, these factors contribute to rising rates of NCDs, including T2DM, hypertension, dyslipidemia, and cardiovascular disease, affecting age groups previously considered at low risk [75,76,77,78,79].

Metabolic exposures during gestation and early childhood appear to play a critical role in shaping long-term susceptibility to insulin resistance, obesity, and other NCDs among children growing up in developing societies [80]. The therapeutic and secondary prevention strategies required to address these conditions and their associated disabilities impose a substantial economic burden on health systems that are often fragile and insufficiently resourced [81,82,83].

Table 5 summarizes the principal public health and policy actions required to prevent childhood obesity, emphasizing context-specific interventions, healthier food environments, life-course prevention, and equity-focused surveillance.

## 7. Strengths and Limitations

Our manuscript is not a systematic review; therefore, we must acknowledge a potential selection and language bias, the absence of formal quality appraisal, and the limitations inherent to a narrative synthesis.

However, we believe our manuscript offers a distinct and varied perspective on excess weight in pediatric subjects by examining it through the lens of socioeconomic conditions. We believe our work offers a different perspective on excess weight in developmental age, helping focus on its health implications across different socioeconomic conditions.

## 8. Conclusions

Childhood obesity is a major and growing global health challenge shaped by the complex interaction of biological, behavioral, environmental, social, and economic factors. Excess adiposity, particularly visceral fat, is associated with early insulin resistance, hypertension, dyslipidemia, hepatic steatosis, and other cardiometabolic abnormalities that increase the long-term risk of metabolic syndrome, type 2 diabetes, and cardiovascular disease.

The burden of childhood obesity and its complications is not distributed equally. Socioeconomic and geographic inequalities influence exposure, access to prevention and treatment, and clinical outcomes. Poverty, stigma, discrimination, limited access to healthcare, unhealthy food environments, and restricted opportunities for physical activity may increase obesity risk and exacerbate its consequences. Parental resources, household food security, food affordability, cultural perceptions of body weight, early growth patterns, school environments, and exposure to unhealthy food marketing further shape dietary behaviors and weight trajectories. These determinants are particularly relevant in low- and middle-income countries undergoing rapid nutritional and socioeconomic transitions.

Effective management therefore requires more than individual lifestyle modification. Early identification of excess weight, systematic screening for comorbidities, and timely, family-centered multidisciplinary care should be integrated with broader interventions addressing food systems, education, healthcare access, and the social and commercial determinants of health. Greater attention to the socioeconomic burden of pediatric obesity is essential for developing equitable, context-specific prevention and treatment strategies. Further research should clarify how socioeconomic conditions influence obesity trajectories, complications, and responses to intervention, thereby supporting more personalized clinical care and better-targeted public health policies.

## Figures and Tables

**Table 1:** The effect of the “4A Framework” on weight excess in pediatric subjects adapted from [14,15,16].

Dimension	Mechanism of Action	Possible Impact on Weight Excess in Pediatric Subjects
Availability	Massive global surge in ultra-processed foods and fast-food chains.	Replaces traditional, whole-food dietary patterns with high-fructose corn syrups and trans fats from the first year of life.
Accessibility	Low-income communities are disproportionately crowded with fast-food outlets and convenience stores while lacking full-service supermarkets (known as “food deserts”).	Children of less-educated mothers live near up to twice as many fast-food outlets, increasing their risk of weight gain as they grow older.
Affordability	Subsidies favor mass-produced agricultural commodities (corn, soy), making unhealthy junk food artificially cheaper than healthy alternatives.	Budgets stretch further by purchasing high-calorie, nutrient-poor items, cementing a social gradient where low-income children experience higher obesity rates.
Appeal	Aggressive digital marketing, colorful packaging, and cartoon mascots target children through television, apps, and video games.	These conditions shape early taste preferences toward hyper-palatable, high-sugar foods, making it incredibly difficult to establish healthy eating habits later.

The overlap of low socioeconomic status and an unhealthy food environment generates a reinforcing feedback loop. Dietary variations arise in the initial year of a child’s life and continue throughout adolescence.

**Table 2:** Clinical management considerations in pediatric obesity adapted from [34,35,36].

Strategy	Description	Objective
Phenotype-based risk stratification	Assessment beyond BMI, including metabolic profile, blood pressure, and fat distribution	Identification of children at highest cardiometabolic risk
Early multidimensional screening	Periodic evaluation of blood pressure, lipid profile, and glycaemic markers, with targeted neurological or dermatological assessment	Early detection of obesity-related complications
Mechanism-based interventions	Lifestyle modification (diet and physical activity), behavioral strategies, and evidence-based pharmacotherapy when indicated	Reduction in visceral adiposity, improved insulin sensitivity, and decreased systemic inflammation

**Table 3:** The socioeconomic gradient of obesity varies across levels of economic development, reflecting the nutrition transition, urbanization, and changing food environments [34,35,36].

Economic Context	SES–Obesity Pattern	Key Determinants
Low-income countries	Higher prevalence among children from higher socioeconomic households	Greater access to calorie-dense foods, urbanization, reduced physical activity
Middle-income countries	Heterogeneous or reversing gradient, with increasing prevalence among lower-SES groups	Expansion and affordability of ultra-processed foods, obesogenic food environments
Regional variation	Positive SES gradient persists in several sub-Saharan African settings, whereas Latin America and parts of Asia show increasing obesity in lower-SES urban populations	Rapid nutrition transition and socioeconomic development

**Table 4:** Overview of Key Studies, Study Designs, Findings, and Literature References Included in the Manuscript Review.

Category/Topic	Study Design & Methodology	Study/Data Source	Sample Size/Cohort Details	Major Findings	References
Global Epidemiology & Forecasting	Global modeling and forecasting study (Global Burden of Disease 2021)	GBD 2021 Adolescent BMI Collaborators (2025)	Pooled global data on youth aged 5–24 years (493 million affected by overweight or obesity in 2021)	Combined prevalence of overweight and obesity doubled, while obesity tripled between 1990 and 2021. Projected 361 million young people aged 5–24 years will be living with obesity by 2050.	[8]
National Surveillance	Epidemiological population-based surveillance system	OKkio alla Salute—Italian National Institute of Health (2023–2025)	Nationally representative sample of Italian children aged 8–9 years	In 2023, 19% of children were overweight, and 9.8% were obese (including 2.6% with severe obesity). Prevalence has plateaued in recent years.	[12,13]
Cardiovascular Risk	Multi-center international observational registry	EAS FH Studies Collaboration (Elshorbagy et al., 2025)	Large cohort of patients with Heterozygous Familial Hypercholesterolemia	Excess weight and obesity independently increase overall cardiovascular disease (CVD) risk and clinical burden.	[5]
Long-Term Mortality & Morbidity	Systematic review of longitudinal cohort studies	Reilly & Kelly (2011)	5 long-term cohort studies	Childhood/adolescent obesity is associated with increased early mortality (HR 1.1 to 5.1) and higher adult cardiometabolic disease incidence.	[13]
Healthcare Service Utilization	Two-cohort prospective study	Hagman et al. (2025)	Two large prospective cohorts followed from adolescence into early adulthood	Adolescent obesity is directly linked to higher healthcare utilization in early adulthood.	[17]
Pathophysiology & CKM Integration	Scientific statement/Clinical consensus review	American Heart Association (Ndumele et al., 2023)	Comprehensive synthesis of clinical and pathophysiological evidence	Establishes Cardiovascular-Kidney-Metabolic (CKM) syndrome; demonstrates how childhood excess adiposity triggers early inflammation, insulin resistance, and subclinical vascular damage.	[39]
Socioeconomic Status Gradient (LMICs)	Systematic reviews and multi-country pooled cohort analyses	Fruhstorfer et al. (2016); Vazquez & Cubbin (2020); NCD-RisC (2024)	Population-based studies across low- and middle-income regions (Sub-Saharan Africa, Latin America, Asia)	Low-income settings: Positive association between higher SES and childhood obesity (greater access to calorie-dense foods). Middle-income settings: Gradient shifts or reverses, with lower-SES groups increasingly exposed to ultra-processed foods.	[55,57,59,61]
Early Clinical Management & Guidelines	Clinical practice guidelines and expert consensus position papers	AAP Guidelines (Hampl et al., 2023); SIEDP/SIP Consensus (Maffeis et al., 2023)	Evidence syntheses of RCTs, observational studies, and expert consensus	Early diagnosis via BMI/waist-to-height ratio, systematic comorbidity screening (lipids, BP, glycemia), and family-centered multidisciplinary care combined with public policies (SSB taxes, marketing restrictions) are critical.	[1,7,29]

**Table 5:** Public health and policy implications, adapted from [84,85,86,87,88,89].

Policy Area	Key Actions
Context-specific prevention strategies	Tailor interventions according to the stage of the nutrition transition within each country
Regulation of food environments	Restrict marketing of unhealthy foods to children, improve school food standards, and implement fiscal measures such as taxes on sugar-sweetened beverages
Life-course prevention	Promote maternal nutrition, breastfeeding, and prevention of rapid weight gain following early growth restriction
Equity-focused surveillance	Monitor childhood obesity by SES indicators, sex, age group, and geographic context to guide targeted interventions

## Data Availability

No new data were created or analyzed in this study. Data sharing is not applicable to this article.

## Abbreviations

The following abbreviations are used in this manuscript:

AAP	American Academy of Pediatrics
BMI	Body Mass Index
CKD	Chronic Kidney Disease
CVD	Cardiovascular Disease
GBD	Global Burden of Disease
LMICs	low- and middle-income countries
MAFLD	metabolic dysfunction-associated fatty liver disease
NAFLD	Non-Alcoholic Fatty Liver Disease
NCDs	Non-communicable diseases
SES	socioeconomic status
T2DM	Type 2 Diabetes Mellitus
